# Efficacy of a docetaxel-5FU-oxaliplatin regimen (TEFOX) in first-line treatment of advanced gastric signet ring cell carcinoma: an AGEO multicentre study

**DOI:** 10.1038/s41416-018-0133-7

**Published:** 2018-06-06

**Authors:** Simon Pernot, Olivier Dubreuil, Thomas Aparicio, Karine Le Malicot, David Tougeron, Céline Lepère, Cedric Lecaille, Lysiane Marthey, Juliette Palle, Jean-Baptiste Bachet, Aziz Zaanan, Julien Taieb

**Affiliations:** 1 Department of GI Oncology, Hôpital Européen Georges-Pompidou, APHP, Paris Descartes University, Sorbonne Paris Cité, Paris, France; 2 Hôpital de la Pitié Salpétrière Hospital, Paris, France; 3 Department of Gastroenterology, Hôpital Saint-Louis, APHP, Paris Diderot University, Sorbonne Paris Cité, Paris, France; 4 Fédération Française de Cancérologie Digestive, Faculty of Medicine, Dijon, France; 5 Department of Gastroenterology, Poiteirs University Hospital, Poitiers, France; 6 Department of Gastroenterology and Digestive Oncology, Polyclinique de Bordeaux Nord, Bordeaux, France; 7 Hôpitaux Universitaires Paris Sud, Gastroenterology Unit, APHP, Paris-Sud University, Le Kremlin Bicêtre, France

**Keywords:** Chemotherapy, Gastric cancer

## Abstract

**Background:**

Triplet chemotherapy, with docetaxel-5FU-oxaliplatin (TEFOX), has yielded promising results in patients with advanced and operable gastric adenocarcinoma. This may prove useful in treating signet ring cell carcinoma (SRCC), which is known to be chemoresistant and has a poor prognosis. We therefore evaluated TEFOX in patients with untreated advanced SRCC.

**Methods:**

Patients with metastatic or locally advanced non-resectable SRCC were treated with TEFOX. Chemotherapy was administered every 14 days, with combined docetaxel (50 mg/m^2^) and oxaliplatin (85 mg/m^2^) followed by 5FU (2400 mg/m^2^).

**Results:**

Among 65 patients enrolled, including 17 with linitis plastica, ORR and DCR were 66.1% and 87.6%, respectively. Median PFS and OS were 9.7 months (95% CI [6.9–11.4]) and 14.3 months (95% CI [11.6–21.6]) respectively. Twenty-six patients (40%) initially considered as unresectable had secondary resection (*n* = 24) or radiotherapy (*n* = 2) with curative intent, with median PFS and OS of 12.4 and 26.2 months, respectively.

**Conclusions:**

TEFOX appears to be effective as first-line treatment in advanced gastric SRCC and has an acceptable safety profile. It allowed a curative intent approach in 40% of patients. Considering the low chemosensitivity of SRCC reported with other chemotherapy regimens and pending for randomised studies, TEFOX might be an option in advanced gastric SRCC.

## Introduction

Gastric cancer is a major public health problem, with 951,000 new cases identified worldwide in 2012, representing 6.8% of all new cancer cases. In 2012, 723,000 patients died of a gastric cancer, accounting for 8.8% of cancer-related deaths.^1^ Despite a decrease in the overall incidence of gastric cancer in recent decades, the incidence of signet ring cell carcinoma (SRCC) is constantly increasing and in recent studies accounts for 35%–45% of gastric adenocarcinoma cases.^2,3^ Its incidence increased 10-fold between 1970 and 2000.^4^ Advanced gastric SRCC is generally thought to have a worse prognosis and lower chemosensitivity than gastric non-SRCC. It therefore remains unclear whether a specific therapeutic strategy is justified, as sensitivity to taxane-based chemotherapy currently remains unclear.

Systemic chemotherapy for locally advanced or metastatic gastric adenocarcinoma is effective in terms of quality of life and survival time.^5^ In many guidelines, as in the European Society for Medical Oncology guidelines,^6^ doublet therapy with fluoropyrimidines and platinum salt (FP) is the reference for palliative chemotherapy in advanced gastric cancer (AGC). However, triplet regimens are a valuable option in fit patients with AGC, in particular taxane-based triplet chemotherapy.

The docetaxel-cisplatin-5FU (DCF) regimen has been shown to be superior to FP^7,8^ in a phase III trial, but has not been widely used because of its poor tolerability. Attempts to improve the therapeutic index of the DCF regimen have been explored in many trials by lowering the doses, modifying the schedule or replacing Fluorouracil (5FU) or cisplatin by better tolerated drugs. Oxaliplatin (O) can advantageously replace cisplatin as shown in several prospective trials,^9,10^ due to a better safety profile. Thus, several trials have tested the triplet combination of D, O and F in various regimens and have shown promising efficacy in AGC. This combination has recently been validated as a new standard (docetaxel, oxaliplatin and 5FU (FLOT) regimen) in operable gastric cancer patients.^11^ This triplet regimen is now a well-accepted option in first-line treatment when intensification of the FP regimen is considered. We previously reported the efficacy of the docetaxel-5FU-oxaliplatin (TEFOX) regimen in AGC and suggested that this regimen could be of value in the subgroup of SRCC and in particular in linitis plastica.^12^

We report here for the first time the evaluation of the triplet TEFOX regimen as first-line chemotherapy in a large cohort of patients with a gastric SRCC.

## Patients and methods

### Patient inclusion

All consecutive patients treated with the TEFOX regimen in seven participating French centres from March 2008 to June 2015 were enrolled in our database. Follow-up data were collected until July 2017.

Eligibility criteria wereas follows: (1) gastric or gastroesophageal junction (GEJ) adenocarcinoma, locally advanced or metastatic, with no possibility of curative resection as assessed by each centre’s multidisciplinary staff including an experienced surgeon; (2) at least one measurable lesion; (3) SRCC according to the World HEalth Organization definition, and categorised as pure SRCC (with 100% SRC) or mixed SRCC (contingent of SRC with at least 50% SRCC but not exclusive); and (4) no previous chemotherapy for advanced disease.

The study was conducted in accordance with the Declaration of Helsinki. All participating patients allowed the use of their medical records for clinical research purposes (if alive at the time of data collection).

### Treatment schedule

The biweekly intravenous TEFOX regimen was given as follows: docetaxel (50 mg/m^2^), oxaliplatin (85 mg/m^2^), and leucovorin (400 mg/m^2^) on day 1, followed by continuous infusion of 5FU for 48 h (2400 mg/m^2^) administered every 2 weeks. Prophylactic treatments, such as corticosteroids, antiemetic, or haematopoietic growth factor, were given according to standard recommendations and to physician’s assessment. Dose reductions and treatment discontinuations were performed according to toxicity, disease progression, and the physician’s decision.

### Outcome and follow-up

#### Response

The objective response rate (ORR) was evaluated every four to six cycles, according to RECIST criteria v1.1 based on a chest, abdomen, and pelvis computed tomoraphy (CT) scan (or magnetic resonance imaging if needed), compared with a baseline CT-scan performed before the first cycle of TEFOX. Disease control rate was defined as the percentage of complete or partial responses or stable disease.

#### Safety

Toxicity was evaluated before each cycle according to the NCI-CTC-AE v4.

### Statistical analysis

Toxicity and ORR were evaluated in the modified intent-to-treat population defined as patients who received at least one cycle of TEFOX. Time to progression was defined as the time elapsed from the start of TEFOX chemotherapy until the date of disease progression. Progression-free survival (PFS) was defined as the time elapsed from the start of TEFOX chemotherapy until the date of progression or death (all causes), whichever occurred first. Alive patients without disease progression were censored at the last follow-up date. Overall survival (OS) was defined as the time elapsed from the start of TEFOX until death (all causes). Alive patients were censored at the last follow-up date. Survival curves were estimated using the Kaplan–Meier method. Median follow-up and its 95% confidence interval (CI) were calculated with the reverse Kaplan–Meier method.

## Results

Sixty-five patients treated with TEFOX were included in this study. Their mean age was 52 years (range, 24–74 years) (Table 1).

**Table 1:** Patient characteristics

All		*N* = 65
Sex	Male	35 (53.8%)
	Female	30 (46.2%)
Age	Mean (SD)	52.35 (11.11)
	Min–max	24–74
Performance status	0	15 (23.1%)
	1	42 (64.6%)
	2	7 (10.7%)
	NE	1 (1.5%)
Localisation	Cardia/GEJ	18 (27.7%)
	Fundus/body	16 (24.6%)
	Pyloric antrum	14 (21.5%)
	Linitis	17 (26.2%)
Histology	Pure SRCC	32 (49.2%)
	Mixed SRCC	33 (50.8%)
Disease stage	Locally advanced	9 (13.8%)
	Metastatic	56 (86.2%)
Metastatic site	Liver only	3 (5.4%)
	Peritoneal carcinomatosis only	28 (50.0%)
	Lung only	1 (1.8%)
	Other, sites with 1 organ involved	9 (16.1%)
	More than 1 organ involved	15 (26.8%)

*GEJ* gastroesophageal junction, *SRCC* signet ring cell carcinoma

A mean of 8.4 cycles of TEFOX were administered per patient (range, 1–29) and 94% of patients received at least 4 treatment cycles (*n* = 62).

### Safety

Toxicities are described in Table 2. Grade 3/4 toxicities occurred in 25 patients (43.5%). There was no treatment-related death. The most common grade 3/4 toxicities were neutropaenia (17.9%), neurotoxicity (16.1%), and nausea (10%). Febrile neutropaenia occurred in one patient (1.9%). Primary prophylactic granulocyte macrophage colony-stimulating factor (G-CSF) was administered to 47 patients (72%). In this group of patients, the rate of grade 3–4 neutropaenia was 12% compared with 28% in patients without prophylactic G-CSF.

**Table 2:** Toxicity (NCI-CTCAE v4.0)

Maximal toxicity	Grade 3	Grade 4
All	40.3%	3.2%
Neutropaenia	14.3	3.6%
Febrile neutropaenia		1.8%
Anaemia	5.3%	0%
Thrombocytopeania	0%	0%
Neurotoxicity	16.1%	
Nausea	10%	0%
Asthenia	5%	0%
Vomiting	3.4%	0%
Mucitis	1.8%	0%
Diarrhoea	1.7%	0%
Edema	3.1%	0%

Adverse events led to treatment discontinuation in 13 patients (20.6%). The causes of interruption were cutaneous reactions considered as grade 2 allergic reactions to docetaxel (*n* = 4), neuropathy (*n* = 6), edaema (*n* = 1), neutropaenia (*n* = 1), and asthenia (*n* = 1).

### Efficacy

**Fig. 1:**
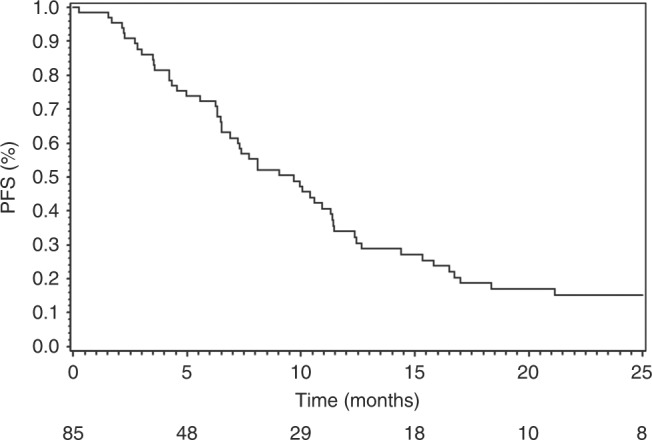
Progression-free survival

**Fig. 2:**
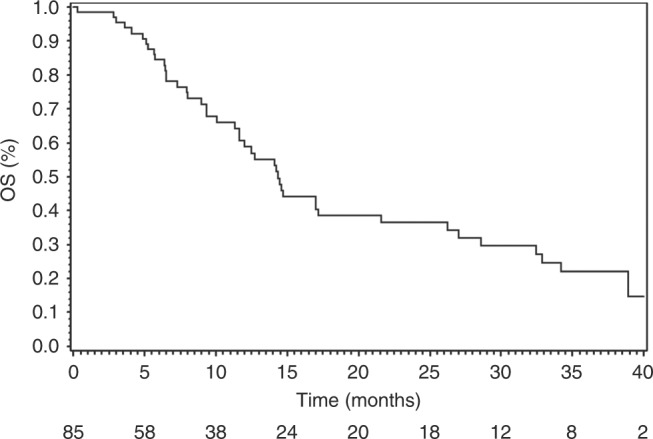
Overall survival

Overall, 43 patients had an objective response, the ORR was 66.1%, and the disease control rate was 87.6%, with 3 confirmed radiological complete responses in 3 metastatic patients after respectively 4, 8, and 9 cycles (Table 3). After a median follow-up of 35.3 months [95% CI (24.6–37.9)], median PFS and OS were respectively 9.7 months (95% CI [6.9–11.4]) (Fig. 1) and 14.3 months (95% CI [11.6–21.6]) (Fig. 2). Median PFS and OS in metastatic patients were respectively 7.4 months (95% CI [6.3–11.4]) and 14.2 months (95% CI [10.1–17.0]). Median PFS in nine LA patients was 10.6 (95% CI [7.7; NR]), and median OS was not reached at the end of follow-up. Overall, no difference was seen in treatment efficacy between patients with pure or mixed SRCC.

**Table 3:** Objective response rate (according to the RECIST v1.1 criteria)

Objective response	*N* (65)	%	95% CI
CR	3	4.6%	[0.9; 12.9]
PR	40	61.5%	[48.6; 73.3]
SD	14	21.5%	[12.3; 33.5]
PD	8	12.3%	[5.5; 22.8]

*CI* confidence interval, *CR* complete response, *PD* progressive disease, *PR* partial response, *SD* stable disease

### Secondary treatment

#### Outcome

Twenty-six patients (40%) received local treatment after response to TEFOX (Table 4 and supplementary fig. 1) after a median of 4 months of induction chemotherapy (range 2.3–13.9).

**Table 4:** Description of secondary local treatment

	*N*	R0
Locally advanced disease (*N* = 9)		
Primary tumour resection	7	6
Radiochemotherapy	2	2 CR
Metastatic disease (*N* = 17)		
PTR + metastasis resection	7	7
PTR after complete regression of metastatic sites	3	3
PTR, PC left	1	0
PTR + RT of metastatic site	3	2
Resection of metachronous metastases	3	2
Total	26	22

*CR* complete response, *PC* peritoneal carcinomatosis, *PTR* primary tumour resection, *RT* radiotherapy

In the 26 patients who underwent local treatment from the start of TEFOX, median PFS and OS were, respectively, 12.4 months [95% CI (10.6–18.4)] and 26.2 months [95% CI = (14.2,NR)], with 2- and 3-year overall survival rates of 51% and 33%. Median disease-free survival after surgery/completion of radiotherapy was 7.3 months [95% CI (6.8–8.9)]. At the end of follow-up, seven patients were alive and relapse-free (four with metastatic disease and three with locally advanced disease) (Supplementary Fig. 1).

#### Locally advanced disease

After an initial response to TEFOX, local treatment was reconsidered in all patients with locally advanced disease. Reconsideration of surgery or local treatment was left to the investigator discretion, considering in most cases the deep response and disappearance of unresecability criteria. Of the nine patients with initially non-resectable locally AGC, seven had an R0 gastrectomy. The two others had GEJ adenocarcinoma and were reconsidered for radiochemotherapy after 3 and 4 months of induction chemotherapy with good response, to improve local control, leading to a complete response in both cases. They received a conformal-three-dimensional radiation therapy, with 50.4 Gy at 18 Gy per day and combined with 5FU-based chemotherapy.

#### Metastatic disease

Among 56 patients with metastatic disease, 17 underwent surgery with curative intent as decided at a multidisciplinary meeting after the response to TEFOX. The initiative of reconsideration of surgical indication was left to the investigator discretion. Seven patients with synchronous metastases underwent primary tumour resection (total gastrectomy or oesophagectomy) combined with resection of metastases (cytoreductive surgery ± hyperthermic intraperitoneal chemotherapy (HIPEC) in four patients with peritoneal carcinomatosis, partial hepatic resection in one, excision of lymph node in two).

Three additional patients underwent resection of the primary tumour plus radiochemotherapy of metastatic sub-clavicular lymph nodes. In three patients, with peritoneal carcinomatosis or metastatic lymph nodes, complete regression of metastatic disease was achieved during surgery and resection of the primary tumour was performed. Three patients with metachronous metastases underwent surgery of the metastatic sites (partial hepatectomy in one and cytoreductive peritonectomy ± HIPEC in two). One additional patient with a macroscopic peritoneal carcinomatosis underwent palliative total gastrectomy.

## Discussion

With a response rate of 65% and median OS of 14 months, the TEFOX regimen administered as first-line treatment for advanced gastric SRCC resulted in a high response rate with an acceptable toxicity profile and allowed secondary resection in 40% of patients even in the metastatic setting.

The chemosensitivity of SRCC is controversial in several reports. In a retrospective study of 924 cases of resected SRCC, comparing patients with and without perioperative chemotherapy (mostly 5FU-platinum doublet ± epirubicin), perioperative chemotherapy was found to be an independent predictor of poor survival (hazard ratio: 1.4, 95% CI [1.1–1.9, *P* = 0.042]) in SRCC patients, possibly due to the toxicities of the neoadjuvant treatment that were correlated with worse post-operative outcome.^13^ However, this study suffers from several biases. The treatment indication and the type of perioperative treatment were left to the investigator’s discretion and patients receiving perioperative chemotherapy had a more aggressive presentation than the others. Similarly, another large retrospective study in a perioperative setting suggested that SRCC had a lower response rate to neoadjuvant chemotherapy.^14^ In this study, response to neoadjuvant treatment was analysed in 723 patients with oesophageal/gastric cancer, 32.5% of whom had SRCC. Among patients with SRCC, 88% received a chemotherapy regimen excluding taxane and 12% a taxane-based regimen. SRCC had a significantly lower clinical to neoadjuvant (21.1% vs. 33.7%, *p* = 0.001) and histopathological response rate (< 10% residual tumour: 16.3 vs. 28.9%, *p* < 0.001) than non-SRCC. However, although the response was less frequent in SRCC, it was associated with improved outcome. Note that, in this study, the use of taxane was significantly associated with better outcome in the whole population, but not in the SRCC group.

In the metastatic setting, there are very few data concerning chemosensitivity in specific subsets of gastric cancer in prospective trials. Twenty years ago, Rougier et al.^15^ reported a 16% response rate in advanced SRCC treated with 5FU + cisplatin, compared with 65% in non-SRCC, suggesting again a limited chemosensitivity of SRCC to common chemotherapeutic regimens.^15^ More recently, a large retrospective analysis of 203 metastatic patients (23% with SRCC) treated with first-line chemotherapy found a response rate in SRCC of 5.3% vs. 28.1% in non-SRCC (*p* = 0.0004). In this study, patients received various regimens based on 5FU/capecitabin and platinum or FOLFIRI in > 80% of cases and only 3% of patients received docetaxel.^16^

As SRCC has specific oncogenic pathways,^17^ it may induce specific sensitivity to targeted agents. There are no data concerning SRCC in recent trials testing targeted agents in gastric cancer. However, efficacy in diffuse type SRCC rather than intestinal type SRCC has been studied in a few trials. In the REGARD trial, ramucirumab, an anti-VEGFR2 antibody, provided a significant benefit in overall survival vs. best supportive care in pretreated patients with gastric cancer.^18^ In subgroup analysis, a significant benefit was found in the diffuse type (hazard ratio 0.56; 95% CI [0.36–0.85]), but not in the intestinal type, suggesting a higher sensitivity to antiangiogenics of diffuse type gastric cancer. This was not found in the RAINBOW trial, which tested ramucirumab in combination with paclitaxel vs. paclitaxel alone,^19^ or in trials with other targeted therapies including anti-HER2.^20^ However, diffuse type was a small subgroup in these trials, and so we cannot draw conclusions regarding specific sensitivity.

Finally, immunotherapy should be tested in SRCC, as PDL1 is overexpressed in about 23% of cases of SRCC, and anti-PDL1/anti-PD1 antibodies are a promising treatment of gastric cancer.^21^ Overall, these results highlight that the classic FP combination or the promising new targeted drugs are suboptimal in conferring a significant survival benefit in gastric SRCC patients in the perioperative or metastatic setting, and that these patients could benefit from intensified and specific treatment.

Recent data suggest that taxane-based therapy could be more effective in SRCC than the classic 5FU-platinum-based regimen. In a retrospective study of resected gastric cancer patients, Chen et al.^22^ found a benefit of docetaxel-based chemotherapy in mixed SRCC compared with oxaliplatin-based chemotherapy. However, the results were conflicting in pure SRCC in which there was no difference between the 2 types of chemotherapy.^22^ In another retrospective study with a limited number of patients with localised SRCC (*n* = 17), docetaxel-based chemotherapy was associated with an 80% R0 resection rate and a median overall survival of more than 40 months.^23^ More recently, the combination of FLOT was tested in a perioperative setting in a phase III randomised trial, and led to a significant benefit as compared with epirubicin, cisplatin and 5FU (ECF) or epirubicin, cisplatin and capecitabin (ECX). In the subgroups of patients with an SRC component, the benefit of FLOT was maintained.^24^

Although surgery was not recommended and left to the investigators discretion, in our study, 40% of patients, considered initially as non-resectable but subsequently as resectable after 2- to 8-month induction chemotherapy, including 9 patients with locally advanced disease and 17 with metastatic disease, had a median OS of 26.2 months, and 33% of them were alive and relapse-free at 3 years. Most investigators considered local treatment indication case by case in particular in case of complete disappearance of macroscopic peritoneal carcinomatosis, or in patients with limited metastatic disease (limited to liver or adenotpahty and to one single metastasis). According to current guidelines, resection of metastatic gastric cancer is not recommended. In a recent publication by the French Surgery Association on 159 metastatic gastric cancer patients whose metastases were resected, median OS remained limited (9.2 months) and only 13% of them were alive at 5 years.^25^ However, some studies report interesting results, in particular in patients with peritoneal carcinomatosis with a strategy combining surgery and HIPEC,^26–28^ but this aggressive management strategy requires experienced teams and centres, together with accurate patient selection because of its limited benefits, high morbidity and mortality. Recent data are, however, encouraging for surgery in patients with limited metastatic disease after effective induction chemotherapy. In a prospective study, 60 patients were treated with neoadjuvant FLOT, a regimen very close to the TEFOX regimen discussed here, before resection of metastatic disease, leading to a median OS of 22.9 months.^29^ In this study, patients were classified as “limited metastatic” before any treatment. Even if these results now have to be confirmed by randomised studies, this report introduces the proof of concept that for a subset of patients with metastatic gastric cancer, resection should be considered. With similar survivals, our results suggest that patients with metastatic gastric SRCC should also be considered for resection of their metastases, when feasible, after a partial response or stability with aggressive systemic induction chemotherapy.

Our study has some limitations. First, our patients were young and had a relatively good general status, as only 10% of them had a performance status of 2. Second, 75% of patients had only one metastatic site. Third, resecability evaluation before treatment was done according to the investigators decision and without defined non-resecability criteria. Some patients were maybe more borderline than unresectable, in particular in locally advanced disease.

In conclusion, whereas SRCC is thought to be less chemosensitive than non-SRCC, recent reports suggest it could have a specific sensitivity profile. Results in this particular subtype may be improved by intensification of treatment using taxane-based chemotherapy. Our study found that TEFOX allowed an excellent control rate and high secondary resection rate and led to prolonged survival in SRCC patients. However, this has to be confirmed in a specific prospective trial.

## Electronic supplementary material

Supplementary figure 1

Supplementary legend

## Availability of data and material

Data are available in FFCD data centre.

## References

[1] Jemal A (2011). Global cancer statistics. CA Cancer J. Clin..

[2] Bamboat ZM (2014). Stage-stratified prognosis of signet ring cell
histology in patients undergoing curative resection for gastric
adenocarcinoma. Ann. Surg. Oncol..

[3] Taghavi S, Jayarajan S, Davey A, Willis A (2012). Prognostic significance of signet ring gastric
cancer. J. Clin. Oncol..

[4] Henson DE, Dittus C, Younes M, Nguyen H, Albores-Saavedra J (2004). Differential trends in the intestinal and diffuse
types of gastric carcinoma in the United States, 1973-2000: increase in the
signet ring cell type. Arch. Pathol. Lab Med..

[5] Wagner AD (2006). Chemotherapy in advanced gastric cancer: a systematic
review and meta-analysis based on aggregate data. J. Clin. Oncol..

[6] Smyth EC (2016). Gastric cancer: ESMO Clinical Practice Guidelines for
diagnosis, treatment and follow-up. Ann. Oncol. J. Eur. Soc. Med. Oncol..

[7] Van Cutsem E (2006). Phase III study of docetaxel and cisplatin plus
fluorouracil compared with cisplatin and fluorouracil as first-line therapy for
advanced gastric cancer: a report of the V325 Study Group. J. Clin. Oncol..

[8] Ajani JA (2007). Clinical benefit with docetaxel plus fluorouracil and
cisplatin compared with cisplatin and fluorouracil in a phase III trial of
advanced gastric or gastroesophageal cancer adenocarcinoma: the V-325 Study
Group. J. Clin. Oncol..

[9] Cunningham D (2008). Capecitabine and oxaliplatin for advanced
esophagogastric cancer. N. Engl. J. Med..

[10] Al-Batran SE (2008). Phase III trial in metastatic gastroesophageal
adenocarcinoma with fluorouracil, leucovorin plus either oxaliplatin or
cisplatin: a study of the Arbeitsgemeinschaft Internistische
Onkologie. J. Clin. Oncol..

[11] Al-Batran SE (2017). Perioperative chemotherapy with docetaxel,
oxaliplatin, and fluorouracil/leucovorin (FLOT) versus epirubicin, cisplatin,
and fluorouracil or capecitabine (ECF/ECX) for resectable gastric or
gastroesophageal junction (GEJ) adenocarcinoma (FLOT4-AIO): A multicenter,
randomized phase 3 trial. J. Clin. Oncol..

[12] Pernot S (2014). Biweekly docetaxel, fluorouracil, leucovorin,
oxaliplatin (TEF) as first-line treatment for advanced gastric cancer and
adenocarcinoma of the gastroesophageal junction: safety and efficacy in a
multicenter cohort. Gastric Cancer.

[13] Messager M (2011). The impact of perioperative chemotherapy on survival
in patients with gastric signet ring cell adenocarcinoma: a multicenter
comparative study. Ann. Surg..

[14] Heger U (2014). Is preoperative chemotherapy followed by surgery the
appropriate treatment for signet ring cell containing adenocarcinomas of the
esophagogastric junction and stomach?. Ann. Surg. Oncol..

[15] Rougier P (1994). Efficacy of combined 5-fluorouracil and cisplatinum in
advanced gastric carcinomas. A phase II trial with prognostic factor
analysis. Eur. J. Cancer Oxf. Engl. 1990.

[16] Lemoine N (2016). Signet ring cells and efficacy of first-line
chemotherapy in advanced gastric or oesogastric junction
adenocarcinoma. Anticancer Res..

[17] Pernot S (2015). Signet-ring cell carcinoma of the stomach: impact on
prognosis and specific therapeutic challenge. World J. Gastroenterol..

[18] Fuchs CS (2014). Ramucirumab monotherapy for previously treated
advanced gastric or gastro-oesophageal junction adenocarcinoma (REGARD): an
international, randomised, multicentre, placebo-controlled, phase 3
trial. Lancet Lond. Engl..

[19] Wilke H (2014). Ramucirumab plus paclitaxel versus placebo plus
paclitaxel in patients with previously treated advanced gastric or
gastro-oesophageal junction adenocarcinoma (RAINBOW): a double-blind, randomised
phase 3 trial. Lancet Oncol..

[20] Bang YJ (2010). Trastuzumab in combination with chemotherapy versus
chemotherapy alone for treatment of HER2-positive advanced gastric or
gastro-oesophageal junction cancer (ToGA): a phase 3, open-label, randomised
controlled trial. Lancet Lond. Engl..

[21] Bang Y.-J., et al. Relationship between PDL1 expression and clinical outcomes in patients with advanced gastric cancer treated with the anti-PD-1 monoclonal antibody pembrolizumab (MK-3475) in KEYNOTE-012. *J. Clin. Oncol.* [cité 28 juin 2015]; Disponible sur: http://meetinglibrary.asco.org/content/150958-156

[22] Chen L (2014). Evaluation of docetaxel- and oxaliplatin-based
adjuvant chemotherapy in postgastrectomy gastric cancer patients reveals obvious
survival benefits in docetaxel-treated mixed signet ring cell carcinoma
patients. Med. Oncol. North. Lond. Engl..

[23] Kim S (2015). The impact of taxane-based preoperative chemotherapy
in gastroesophageal signet ring cell adenocarcinomas. J. Hematol. Oncol..

[24] Al-Batran SE (2016). Histopathological regression after neoadjuvant
docetaxel, oxaliplatin, fluorouracil, and leucovorin versus epirubicin,
cisplatin, and fluorouracil or capecitabine in patients with resectable gastric
or gastro-oesophageal junction adenocarcinoma (FLOT4-AIO): results from the
phase 2 part of a multicentre, open-label, randomised phase 2/3
trial. Lancet Oncol..

[25] Glehen O (2010). Peritoneal carcinomatosis from gastric cancer: a
multi-institutional study of 159 patients treated by cytoreductive surgery
combined with perioperative intraperitoneal chemotherapy. Ann. Surg. Oncol..

[26] Glehen O (2004). Cytoreductive surgery and intraperitoneal
chemohyperthermia for peritoneal carcinomatosis arising from gastric
cancer. Arch. Surg. Chic..

[27] Glehen O (2010). Toward curative treatment of peritoneal carcinomatosis
from nonovarian origin by cytoreductive surgery combined with perioperative
intraperitoneal chemotherapy: a multi-institutional study of 1,290
patients. Cancer.

[28] Yonemura Y (1996). Effects of intraoperative chemohyperthermia in
patients with gastric cancer with peritoneal dissemination. Surgery.

[29] Al-Batran SE (2017). Effect of neoadjuvant chemotherapy followed by
surgical resection on survival in patients with limited metastatic gastric or
gastroesophageal junction cancer: the AIO-FLOT3 Trial. JAMA Oncol..

