# Supplementary figures and images for: Efficacy of a docetaxel-5FU-oxaliplatin regimen (TEFOX) in first-line treatment of advanced gastric signet ring cell carcinoma: an AGEO multicentre study
Source: Br J Cancer. 2018 Jun 6;119(4):424–8. doi: 10.1038/s41416-018-0133-7 (PMC6133962; doi:10.1038/s41416-018-0133-7)

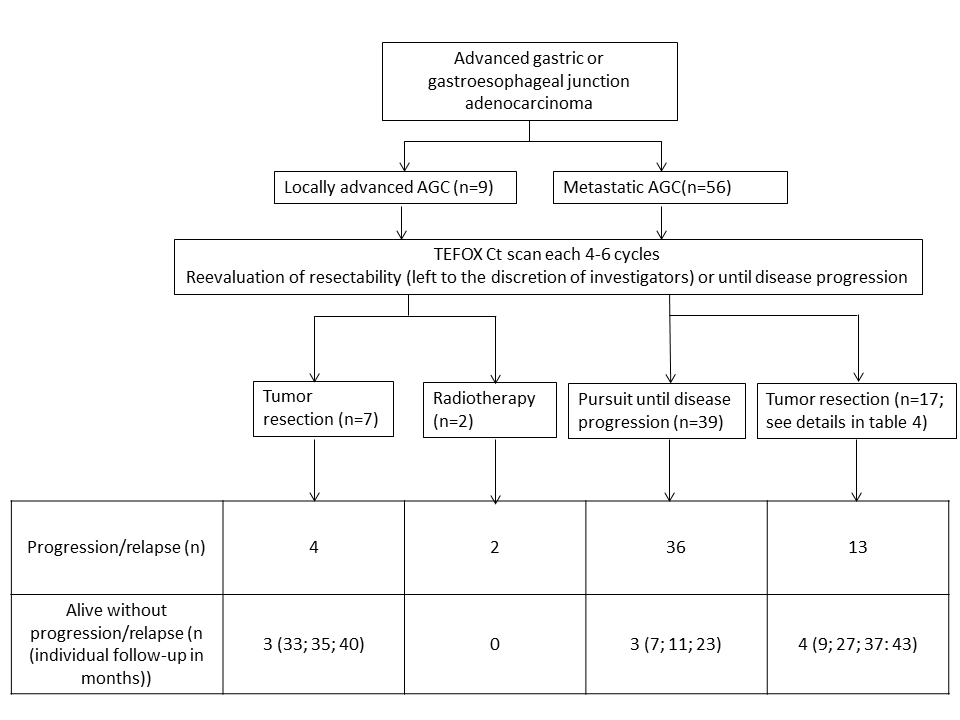

Supplement: Supplementary file 1 — Supplementary figure 1 [file 41416_2018_133_MOESM1_ESM.tif]
