# Supplementary material for: Efficacy of a docetaxel-5FU-oxaliplatin regimen (TEFOX) in first-line treatment of advanced gastric signet ring cell carcinoma: an AGEO multicentre study
Source: Br J Cancer. 2018 Jun 6;119(4):424–8. doi: 10.1038/s41416-018-0133-7 (PMC6133962; doi:10.1038/s41416-018-0133-7)
Supplement: Supplementary file 2 — Supplementary legend [file 41416_2018_133_MOESM2_ESM.docx]

Supplementary Figure 1. Flowchart of approach and outcome of patients
